# Phylogeny meets ecotoxicology: evolutionary patterns of sensitivity to a common insecticide

**DOI:** 10.1111/j.1752-4571.2011.00237.x

**Published:** 2012-01-23

**Authors:** John I Hammond, Devin K Jones, Patrick R Stephens, Rick A Relyea

**Affiliations:** 1 Department of Biological Sciences, University of PittsburghPittsburgh, PA, USA; 2 Odum School of Ecology, University of GeorgiaAthens, GA, USA

**Keywords:** amphibian decline, anuran, contaminant, ecotoxicology, nontarget

## Abstract

Pesticides commonly occur in aquatic systems and pose a substantial challenge to the conservation of many taxa. Ecotoxicology has traditionally met this challenge by focusing on short-term, single-species tests and conducting risk assessments based on the most sensitive species tested. Rarely have ecotoxicology data been examined from an evolutionary perspective, and to our knowledge, there has never been a phylogenetic analysis of sensitivity, despite the fact that doing so would provide insights into patterns of sensitivity among species and identify which clades are the most sensitive to a particular pesticide. We examined phylogenetic patterns of pesticide sensitivity in amphibians, a group of conservation concern owing to global population declines. Using the insecticide endosulfan, we combined previously published results across seven species of tadpoles and added eight additional species from the families Bufonidae, Hylidae, and Ranidae. We found significant phylogenetic signal in the sensitivity to the insecticide and in the existence of time lag effects on tadpole mortality. Bufonids were less sensitive than hylids, which were less sensitive than the ranids. Moreover, mortality time lags were common in ranids, occasional in hylids, and rare in bufonids. These results highlight the importance of an evolutionary perspective and offer important insights for conservation.

## Introduction

Pesticide use can offer economic benefits by controlling pest species and increasing crop yields, but it can also lead to aquatic contamination because of direct overspray, drift, atmospheric transport, agricultural and residential runoff, misuse, and improper disposal (Harris et al. 1998a; McConnell et al. 1998; LeNoir et al. 1999; Wan et al. 2005; Gilliom and Hamilton 2006). For example, surveys done by the U.S. Geological Survey across 51 major river basins and groundwater found at least one pesticide at every sample site (Gilliom and Hamilton 2006). This contamination creates challenges in understanding the population and community effects on nontarget taxa (Harris et al. 1998a; Rohr and Crumrine 2005; Boone et al. 2007; Relyea and Diecks 2008; Relyea and Hoverman 2008; Rohr et al. 2008; Relyea 2009).

The most common approach to determine potential lethal effects on nontarget taxa is to conduct single-species experiments over short time frames (e.g., 1- to 4-day exposures) to generate dose–response curves and estimate LC50 values (i.e., the pesticide concentration that causes 50% mortality). These tests typically focus on a few model organisms (i.e., a bird, mammal, fish, and invertebrate) as part of the registration process and attempt to identify the most sensitive species within each group. The underlying assumptions are species have very little intraspecific variation in sensitivity, hence any population is a good estimate of the species as a whole, and that by estimating the impact on a few sensitive species, one can extrapolate the impact on all species within the group and, in some cases, other distantly related groups. While this approach has been helpful in estimating the potential direct impacts of pesticides, it leaves large gaps in our understanding of species and population sensitivities. In some cases, species within a group have very similar sensitivities to a given pesticide (e.g., invertebrates Ashauer et al. 2011; amphibians Relyea and Jones 2009), while in other cases, species within a group as well as populations within a species can differ in sensitivity by several orders of magnitude (e.g., invertebrates, Wan et al. 2005; Ashauer et al. 2011; amphibians species, Jones et al. 2009; amphibian species and populations, Bridges and Semlitsch 2000). When sensitivity varies widely among species, it would be helpful to know whether populations within a species have similar sensitivities, whether there are patterns in sensitivity that may guide *a priori* predictions about which species are more likely to be affected by a pesticide exposure, and which pesticide might be responsible if only some species within a group are declining. These questions are best answered by incorporating an evolutionary perspective across large scales.

A successful method of investigating large-scale evolutionary patterns in species traits is to take a comparative approach. A comparative approach overlays a species’ trait values onto phylogenetic trees to determine whether species possess similar traits attributable to a shared history or convergence (e.g., Felsenstein 1973; Winermiller 1991; Richman and Price 1992; Schluter 1994; Losos 1996; Losos et al. 1998; Pagel 1999; McPeek and Brown 2000; Richardson 2001; Stephens and Wiens 2004). This approach has rarely been used in toxicology studies and usually has compared only a few closely related species (e.g., Strumbaurer et al. 1999). This is despite the fact that phylogenetic inference would be useful in addressing many ecotoxicological questions including which species are at greatest risk should contamination occur in an area and which contaminant is most likely to be responsible for causing the decline of certain species when habitats become contaminated and only a few of the species in a clade decline.

While taking a phylogenetic approach to pesticide sensitivity would be insightful for many organisms, amphibians are of particular interest. Amphibians are experiencing worldwide population declines, and pesticides have been implicated as one of several potential causes (Alford and Richards 1999; Blaustein and Kiesecker 2002). Pesticides can have substantial lethal effects on individuals (e.g., Relyea and Jones 2009); they can also cause increased sensitivity to parasites (Rohr et al. 2008), reduced growth and survival through community interactions (Relyea and Diecks 2008), synergistic effects through multiple stressors (Taylor et al. 1999) and mixtures (Hayes et al. 2006), have breakdown products that can be more toxic than the parent compounds (Sparling and Fellers 2007), can be endocrine disruptors potentially leading to feminization of males (Hayes et al. 2002), and can bioaccumulate in individuals (Hopkins et al. 1998). These toxic effects of pesticides can happen over short to long temporal scales.

Our knowledge of amphibian sensitivity to most pesticides is poor because the registration of most pesticides does not require any amphibians to be tested. Instead, the sensitivity of aquatic stages of amphibians is estimated from studies of fish (Jones et al. 2004). The underlying assumption is that setting maximum contamination levels in nature, based on the most sensitive fish species, simultaneously protects aquatic amphibians. Hence, there is a substantial need to conduct toxicity tests on amphibians, over both short and long terms, particularly for those pesticides for which there is evidence of high toxicity.

Recent studies have discovered that one of the most toxic pesticides for amphibians is the insecticide endosulfan. Endosulfan can cause high rates of mortality at extremely low concentrations (i.e., <1 ppb), and the mortality differs widely among amphibian species (Berrill et al. 1998; Jones et al. 2009; Relyea 2009). For example, 6 ppb of endosulfan added to wetland mesocosms caused 84% mortality in *Rana pipiens* tadpoles but had no effect on *Hyla versicolor* tadpoles (Relyea 2009). In a study designed to quantify the LC50 values across a range of species, Jones et al. (2009) found that amphibian LC50_4-d_ estimates range from 0.9 to 112 ppb and more closely related species seem to share similar sensitivities. Moreover, the researchers found that, for several tadpole species, endosulfan caused substantial lag effects between the time of exposure and the time of death. Unlike toxic effects attributed to factors like bioaccumulation (e.g., Hopkins et al. 1998) or endocrine disruption (e.g., Hayes et al. 2002), endosulfan can cause death of tadpoles several days after the exposure has ended (Jones et al. 2009). Collectively, these studies suggest that there may be phylogenetic patterns in the sensitivity of amphibians to endosulfan and in the existence of mortality lag effects.

Here, we expanded the original set of LC50 values for seven species estimated by Jones et al. (2009) to 15 species across the Bufonidae, Hylidae, and Ranidae families to permit a phylogenetic analysis of amphibian endosulfan sensitivity. For one species (*Rana sylvatica*), we also examined endosulfan sensitivities among six populations to estimate within-species variation in sensitivity and help interpret the amount of variation observed within versus among species. We tested the following hypotheses: (i) amphibian populations will differ in their sensitivity to endosulfan; (ii) amphibian species will differ in their sensitivity to endosulfan; (iii) amphibian species will differ in their occurrence of mortality lag effects; and (iv) differences in LC50 values and mortality lag effects will exhibit phylogenetic patterns.

## Materials and methods

### Pesticide background

Endosulfan is an organochlorine insecticide that excites the neuromuscular system, can damage eye and gill tissues, and is a potential endocrine disruptor (Harris et al. 2000; Rossi 2002; Bernabò et al. 2008). Example application rates on crops such as corn, cotton, and lettuce average approximately 1.2 kg/ha in California USA during 2008 (http://www.pesticideinfo.org/Detail_ChemUse.jsp?Rec_Id=PC35085). Expected aqueous concentrations range from 700 ppb in pond water 10 m away from targeted application sites to 4 ppb in pond water 200 m away (sprayed from a nozzle 3–4 m above the ground; Ernst et al. 1991). It has been reported at 0.5 ppb in ponds near apple orchards in Ontario, Canada (Harris et al. 1998a), 2.5 ppb in Australian aquatic environments (Muschal 2001), and the US EPA (Rossi 2002) estimated the range of expected environmental concentration (EEC) for surface drinking water at 0.5–23.9 ppb. Endosulfan has also been detected in surveys of amphibians and fish tissues and is highly toxic (i.e., 100 < LC50 < 1000 ppb) or very highly toxic (i.e., LC50 < 100 ppb) to fish, amphibians, and crustaceans (Berrill et al. 1998; Leight and Van Dolah 1999; Sparling et al. 2001; Rossi 2002; Wan et al. 2005; Jones et al. 2009; Relyea 2009).

#### Species-level variation experiment

All animals were collected as newly oviposited egg masses from around the United States. Species from California, Florida, North Carolina, Oregon, and Tennessee were shipped overnight to the University of Pittsburgh’s Pymatuning Laboratory of Ecology in ice chests containing cold packs. Species from Michigan and Pennsylvania were collected and driven to the laboratory. Egg masses were hatched in covered outdoor culture pools containing well water, and the hatchlings were fed daily with rabbit chow (*ad libitum*).

To examine the lethality of endosulfan to tadpoles, we used the methods detailed in the study by Jones et al. (2009). In brief, we exposed each species to a range of concentrations over a 4-day period to quantify traditional LC50_4-d_ values. We subsequently moved the tadpoles from all treatments into clean water for an additional 4-day to estimate potential lag effects. For each species, except *Rana aurora*, we employed a randomized block design that contained two blocks (laboratory shelf heights) and two replicates of each treatment per block. Treatments included a negative control (water), a vehicle control (ethanol) due to endosulfan’s low solubility in water, and six nominal concentrations of endosulfan (1, 5, 10, 50, 100, and 500 ppb). The amount of ethanol added for the vehicle control was set to equal the amount of ethanol in the 500 ppb endosulfan concentration treatment (ethanol concentration = 0.5%). For *R. aurora*, we had fewer individuals available and therefore replicated each treatment three times using a single experimental block. All species were of similar sizes or similar developmental stages (Gosner 1960) (Appendix Table A2).

For each species, tadpoles were tested as groups of 10 animals held in 1-L plastic containers filled with 500 mL of carbon-filtered, UV-irradiated well water. The water was changed daily and the pesticide was reapplied. Large volumes of each concentration solution (2.75 L) were prepared each day by adding different amounts of a concentrated stock solution (0.1 mg a.i./mL; technical-grade endosulfan, 99% purity Chem Services Inc., West Chester, PA, USA) to separate containers and then distributing these nominal solutions to the appropriate experimental units. To achieve nominal concentrations of 1, 5, 10, 50, 100, and 500 ppb, we added 27.5, 137.5, 275, 1375, 2750, and 13 750 μL of the stock solution to the 2.75 L of water. At 24-h intervals, we quantified survival, removed any dead individuals, and changed the water. Prior to the 48-h water change, we measured temperature (across all species temperature ranged from 17.3 to 21.9°C; within each species experimental units ranged 0.5–1.6°C) and pH (across all species ranged from 8.0 to 8.5).

For each species at each of the four exposure water changes, we collected a 125 mL sample of each nominal concentration in a precleaned glass amber jar and mixed them together to create 500 mL per exposure level per species. To analyze the concentration of endosulfan used in the eight, single-species experiments, we pooled the samples across five species, then across two species, and finally across the remaining single species. The three sets of pooled samples were shipped to the Mississippi State Chemical Laboratory (Mississippi State, MS) for independent analysis (excluding the ethanol vehicle controls). For the five-species pooled sample (*Bufo americanus*, *R. pipiens*, *R. aurora*, *R. sphenocephala*, and *R. sylvatica)*, actual endosulfan concentrations were 0.5, 1.7, 3.2, 20.2, 38.0, and 162.0 ppb (henceforth referred to as 0.5, 2, 3, 20, 38, and 162). For the two-species pooled sample (*Pseudacris feriarum* and *Pseudacris triseriata*), actual endosulfan concentrations were 0.4, 0.9, 2.1, 14.3, 17.5, and 109.2 ppb (henceforth referred to as 0.4, 1, 2, 14, 18, and 109 ppb). For the final water sample (*Rana boylii*), actual endosulfan concentrations were 0.8, 2.5, 3.2, 16.4, 38.0, and 145.4 ppb (henceforth referred to as 1, 2, 3, 16, 38, and 145). No endosulfan was detected in any control.

After the initial 4-day exposure, all animals were transferred to clean water for an additional 4 days. During this postexposure period, we changed the water every 24 h and fed the tadpoles a 2% mean per-capita ration of ground Tetramin fish flakes (Tetra, Blacksburg, VA, USA). Daily mortality checks continued every 24 h. After 8 days, we recorded survival and euthanized the remaining tadpoles in 2% MS-222. Animal bodies and experimental water were disposed of in accordance with university protocols for environmental health and safety.

#### Population-level variation experiment

Next, we investigated variation in sensitivity among six populations of *R. sylvatica*. Five of the populations were from northwestern Pennsylvania and one was from Eastern Michigan. All populations were at least 20 km apart, which generally assures population differentiation (Newman and Squire 2001). We used the same methods described above with the following changes. We used alterations of temperature (by moving eggs between the indoor laboratory and outdoor ambient air) to cause the eggs from all populations to hatch within 24 h. By standardizing development time post hatching across all populations, we removed any effect of age which can alter sensitivities (e.g., Jones et al. 2010). This allowed us to include the natural variation in development and growth rate commonly found in *R. sylvatica* populations (Relyea 2002), and allowed us the ability to test all populations at the same time. For each population, we transferred 200 hatchlings to 90-L pool containing well water. The hatchlings were fed 5 g of rabbit chow weekly until the start of the experiment (see Appendix Table A3 for initial tadpole mass and developmental stage).

We used nominal concentrations of 10, 50, 100, and 500 ppb and renewed the treatments daily. We did not employ an ethanol vehicle control due to the size of the experiment and because our single-species experiments generally show little difference between the water controls and ethanol controls. Each day, we quantified survival, removed any dead individuals, and monitored temperature (range = 19.7–20.2°C) and pH (range = 8.0–8.3). The experiment was ended after a 2-day exposure, rather than a 4-day exposure, because the lower concentrations were showing increasing mortalities with the higher concentrations showing 100% mortality. To generate LC50 estimates with confidence intervals, at least two concentrations are needed with nonzero or 100% mortality.

At each water change, 500 mL was sampled and placed in a precleaned glass amber jar for testing. To determine the actual concentrations, we pooled samples across both days and shipped them for independent analysis (see above). Actual concentrations were 5.3, 23.3, 54.0, and 287.0 ppb (henceforth referred to as 5, 23, 54, and 287) with no detectable endosulfan in the control.

### Statistical analysis

#### Species-level variation experiment

To determine how the range of endosulfan concentrations affected tadpole survival in the single-species experiments, we used the proportion of individuals surviving in an experimental unit as our response variable. Because of low variance in the lowest and highest concentrations (i.e., either 100% or 0% survival across all replicates, respectively), we rank-transformed the survival data and used these ranks as the response variable in a repeated-measures analysis of variance (rm-anova). Our repeated measure was time and ranged from day 1 to 8. Next, we tested for the lowest observable effect concentration (LOEC). There are many logically ways of comparing the controls to the pesticide gradient, in this case the two control treatments were never significantly different in survival (*P* > 0.10). Hence, we chose to use one-tailed Dunnett’s tests to compare survival in the water control and each endosulfan concentration treatment at the 4- and 8-day time points.

To estimate the LC10, LC50, and LC90 estimates at 4- and 8-day intervals, we used probit analyses to fit a sigmoid-shaped curve to the data after adjusting for control mortality (smoothing the average mortality and using Abbott’s formula; Finney 1971). We also did this for the seven species reported by Jones et al. (2009). In three cases, this produced 8-day concentration mortalities lower than their 4-day counterparts as a result of differences in mortality in the controls. We adjusted the three 8-day estimates back to their 4-day estimates to reflect no decrease in mortality. On the basis of these LC50 estimates, we tested whether the 4- and 8-day estimates were significantly different by comparing the overlap between the 84% confidence intervals. Simulation tests have shown this method approximates an α = 0.05 (Payton et al. 2003).

#### Population-level variation experiment

We used similar methods to the single-species experiments except the data focused on the proportion of individuals surviving on day 2. Given there was no mortality in the controls, no adjustments were needed to generate LC50 estimates.

#### Phylogenetic analysis

Phylogenetic signal can be defined as a pattern of trait variation whereby more closely related species have more similar traits (i.e., trait disparity among species is correlated with phylogenetic distance). We tested for phylogenetic signal in pesticide susceptibility using a standard measure of phylogenetic signal: Blomberg’s *K* (Blomberg et al. 2003). Blomberg’s *K* is defined as the ratio of observed phylogenetic dependence to the phylogenetic dependence expected under a Brownian motion model of character evolution (Blomberg et al. 2003). It varies between zero and ∞, with values of zero indicating phylogenetic independence, values <1 indicating weaker phylogenetic dependence (i.e., weaker signal) than expected under Brownian motion, and values >1 indicating greater phylogenetic dependence than expected.

**Figure A1:**
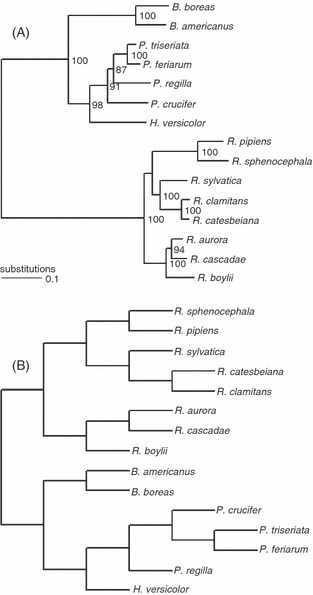
(A) Tree with highest likelihood from a maximum likelihood analysis of mitochondrial sequence data (outgroup not shown). Numbers to the right of each node indicate the proportion of 1000 bootstrap pseudoreplicates that each clade occurred in; values of <50% are not shown and (B) Equal-length-branch tree assembled based on previous studies of anuran phylogeny.

Blomberg’s *K* was calculated for LC50_4-d_ and LC50_8-d_ estimates using the software package Picante v1.2 (Kembel et al. 2010), implemented in R v2.10.1. Two phylogenetic trees were used to quantify phylogenetic distance among species. One tree was based on previous studies of anuran phylogeny and assumed a speciational model of trait evolution (i.e., all branch lengths were set to one), and the other tree was estimated via maximum likelihood from 2500 bp of mitochondrial sequence data obtained from GenBank (see Appendix Fig. A1). The methods used to create these two trees are detailed in Appendix A1. To determine whether values of Blomberg’s *K* for pesticide sensitivity were statistically significant, we performed a permutation procedure implemented in Picante where the variance of phylogenetically independent contrasts of observed data is compared with the variance of contrasts estimated from data shuffled randomly across the tips of the tree (Blomberg and Garland 2002; Kembel et al. 2010). Each test used 10 000 random permutations of the data.

To determine whether the time lag showed significant phylogenetic signal, we used the method of Maddison and Slatkin (1991) where the number of evolutionary transitions a character shows when mapped onto a tree is compared with number of the transitions it shows when shuffled randomly across the tips of a tree and reconstructed. By shuffling a character hundreds of times, it is possible to generate a null distribution of the number of evolutionary steps expected when the character states observed among a set of species vary independently of phylogeny. The presence or absence of a statistically significant difference between LC50_4-d_ and LC50_8-d_ values, based on comparing 84% confidence intervals in LC50 in each species, was coded as a discrete character: (0), no significant time lag (i.e., confidence intervals overlapped); (1), significant time lag (confidence intervals do not overlap). The presence or absence of a time lag was then reconstructed for interior nodes using maximum likelihood (Schluter et al. 1997). The number of evolutionary steps exhibited by the time lag was calculated by counting the number of branches on which the character state with the highest likelihoods differed between an ancestral and descendant node, or where the state with the highest likelihood on an ancestral node differed from the character state assigned to a tip taxon. The time lag was then shuffled randomly without replacement among tips 1000 times, repeating the likelihood reconstruction and counting up the number of evolutionary steps during each randomization.

## Results

### Species-level variation experiment

**Figure 1:**
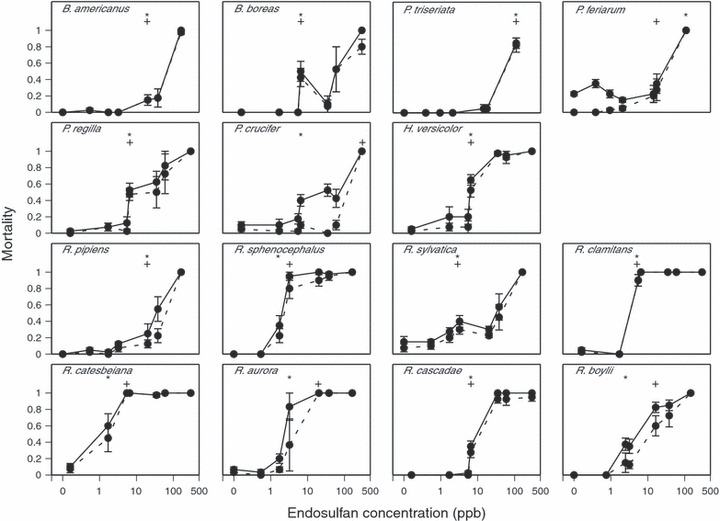
The mortality (means ± 1 SE) of tadpoles when exposed to a range of endosulfan concentrations. Dotted lines indicate mortality after a 4-day exposure to different endosulfan concentrations. Solid lines indicate mortality after a 4-day exposure to different endosulfan concentrations followed by 4-day exposure in clean water. The lowest observable effect concentration (LOEC; compared to the control) is indicated by a plus (+) for mortality after 4 days and an asterisk (*) for mortality after 8 days. Species underlined are from a previous study (Jones et al. 2009).

We began by examining the effects of different endosulfan concentrations on the survival of eight species of tadpoles. Generally, the control treatments had excellent survival within each species (above 93% survival after 4 and 8 days) with only one species, *P. feriarum*, having lower survival (83%) and only after 8 days (see Appendix 2A). The rm-anovas for all species indicated significant effects of endosulfan concentration, time, and their interaction (all *P*-values <0.05) with no block effect on survival (all *P*-values >0.13). Endosulfan significantly reduced survival in all species, and its effect was dose-dependent (Table 1, Fig. 1).

**Table 1:** The estimated LC10, LC50, and LC90 values (with 84% confidence intervals) for 15 species of tadpoles. Estimates were made after a 4-day exposure to a range of endosulfan concentrations followed by a subsequent exposure to 4-day of clean water. All units are in ppb. Species in boldface font have significantly different 4- and 8-day LC50 estimates. Underlined species are estimates from a previous study using the same methods (Jones et al. 2009)

Species	LC10_4-d_	LC50_4-d_	LC90_4-d_	LC10_8-d_	LC50_8-d_	LC90_8-d_
*Bufo boreas*	7.0 (4.4, 10.0)	67.7 (52.2, 91.0)	653 (399, 1296)	7.1 (4.8, 9.7)	43.4 (35.1, 54.6)	264 (184, 432)
*Bufo americanus*	19.6 (14.6, 24.2)	55.5 (46.9, 67.2)	157 (119, 236)	20.1 (15.2, 24.5)	53.0 (45.1, 63.8)	140 (107, 207)
*Pseudacris triseriata*	22.0 (16.3, 27.7)	56.4 (46.4, 69.1)	144 (112, 203)	21.7 (16.2, 27.2)	53.6 (44.2, 65.7)	132 (103, 185)
*Pseudacris feriarum*	2.9 (1.8, 4.2)	25.5 (21.3, 31.4)	88.6 (64.1, 144)	2.9 (1.8, 4.2)	25.3 (19.5, 33.8)	218 (135, 430)
*Pseudacris regilla*	2.7 (1.8, 3.8)	21.3 (17.0, 26.9)	165 (114, 271)	1.7 (1.1, 2.4)	13.9 (11.1, 17.5)	114 (79.0, 187)
***Pseudacris crucifer***	22.0 (15.1, 29.1)	112 (89.4, 146)	571 (383, 1000)	0.8 (0.4, 1.4)	26.0 (18.9, 37.0)	823 (420, 2120)
***Hyla versicolor***	2.4 (1.7, 3.0)	9.0 (7.6, 10.7)	33.8 (25.9, 48.4)	1.1 (0.7, 1.5)	6.0 (4.9, 7.3)	33.2 (24.5, 50.0)
***Rana pipiens***	13.0 (9.0, 17.0)	51.0 (42.1, 63.1)	199 (143, 322)	4.4 (2.8, 6.1)	28.0 (22.3, 35.4)	178 (122, 297)
***Rana sphenocephala***	0.7 (0.5, 0.9)	2.8 (2.3, 3.4)	11.4 (8.5, 17.2)	1.0 (0.8, 1.2)	1.9 (1.7, 2.1)	3.6 (3.1, 4.6)
*Rana sylvatica*	2.6 (1.5, 3.9)	31.4 (23.8, 42.6)	379 (226, 779)	2.0 (1.5, 3.9)	25.1 (19.1, 33.7)	312 (189, 626)
*Rana clamitans*	2.0 (1.6, 2.4)	3.3 (2.8, 3.7)	5.2 (4.6, 6.1)	1.7 (1.4, 2.1)	3.1 (2.7, 3.5)	5.4 (4.7, 6.4)
*Rana catesbeiana*	0.4 (0.2, 0.6)	1.3 (1.0, 1.6)	4.4 (3.5, 5.9)	0.2 (0.1, 0.3)	0.9 (0.7, 1.2)	3.6 (2.8, 5.0)
***Rana aurora***	2.3 (1.6, 2.6)	3.7 (3.3, 4.8)	6.1 (4.7, 12.6)	1.6 (1.3, 1.8)	2.4 (2.2, 2.7)	3.7 (3.2, 4.5)
***Rana cascadae***	4.8 (3.6, 5.9)	15.0 (12.7, 17.9)	47.4 (37.4, 64.0)	6.0 (5.5, 6.1)	6.7 (6.5, 7.1)	7.6 (7.1, 9.0)
***Rana boylii***	2.5 (1.7, 3.3)	13.0 (10.6, 16.1)	67.6 (49.6, 101)	0.9 (0.6, 1.3)	5.4 (4.4, 6.6)	30.6 (22.1, 46.9)

Next, we explored species-level variation in LC50 estimates. After 4 days of exposure, *R. pipiens*, *B. americanus*, and *P. triseriata* had LC50_4-d_ estimates of 50–55 ppb, while *R. sylvatica* and *P. feriarum* had LC50_4-d_ estimates of 25–35 ppb. The most sensitive species were *R. boylii*, *R. aurora*, and *R. sphenocephala* with LC50_4-d_ estimates below 15 ppb.

Finally, we addressed whether each species experienced a lag effect in mortality (i.e., postexposure mortality). After 4 days of exposure followed by 4 days in clean water, *B. americanus* and *P. triseriata* had LC50_8-d_ estimates of 50–55 ppb, while *R. pipiens, R. sylvatica*, and *P. feriarum* had LC50_8-d_ estimates of 25–30 ppb (Table 1, Fig. 1). The most sensitive species were *R. boylii*, *R. aurora*, and *R. sphenocephala* with LC50_8-d_ estimates below 6 ppb. On the basis of 84% confidence of these LC50_8-d_ estimates, four of the eight species (*R. sphenocephala*, *R. pipiens*, *R. aurora*, and *R. boylii*) experienced significant lag effects.

### Population-level variation experiment

**Figure 2:**
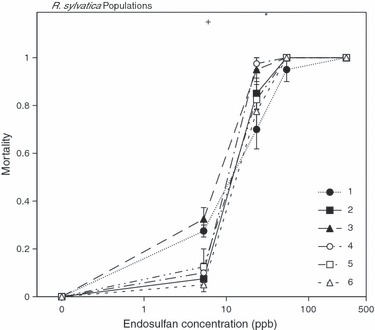
The mortality (means ± 1 SE) of *Rana sylvatica* tadpoles from six populations when exposed to a range of endosulfan concentrations. The lowest observable effect concentration (LOEC; compared to the control) is marked with a plus (+) for populations 1 through 4 and an asterisk (*) for populations 5 and 6.

The anovas for all six *R. sylvatica* populations indicated significant effects of endosulfan concentration (all *P*-values <0.01). For populations 1 through 4, a 2-day exposure to endosulfan increased mortality relative to controls at all concentrations of the insecticide. For populations 5 and 6, only the 23, 54, and 287 ppb concentrations caused significantly more death than the control (Fig. 2). All populations had 100% survival in the controls.

The LC50 estimates range from 13.2 to 23.5 ppb (Table 2). On the basis of 84% confidence intervals, only the most and least sensitive populations were significantly different from each other. Thus, these populations tend to have similar sensitivities to endosulfan, and any single population’s estimate appears to be a good indicator for the other populations.

**Table 2:** The estimated LC10_2-d_, LC50_2-d_, and LC90_2-d_ values (with 84% confidence intervals) for six populations of *Rana sylvatica* tadpoles. All units are in ppb

Population	LC10_2-d_	LC50_2-d_	LC90_2-d_
1	2.4 (1.2, 3.7)	13.2 (10.0, 16.8)	73.5 (52.6, 4119)
2	6.1 (4.3, 7.9)	14.6 (12.1, 17.3)	34.7 (28.7, 45.2)
3	4.5 (2.8, 6.2)	15.9 (12.9, 19.3)	56.1 (43.4, 80.5)
4	9.6 (6.5, 12.1)	19.7 (16.6, 22.5)	40.3 (34.2, 51.8)
5	6.6 (4.4, 8.6)	17.4 (14.4, 20.5)	45.9 (37.3, 61.3)
6	5.6 (3.4, 7.9)	23.5 (19, 28.7)	98.3 (72.9, 152)

### Phylogenetic analysis

**Figure 3:**
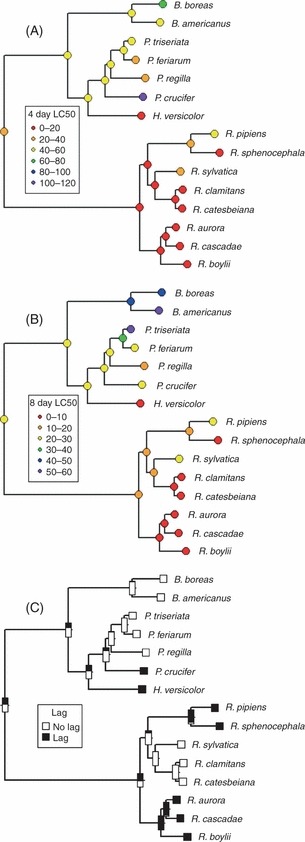
Maximum likelihood reconstruction of the mtDNA tree for (A) LC50_4-d_ estimates, (B) LC50_8-d_ estimates (please note the change in scale), and (C) presence and absence of statistically significant time lag (area of each color is proportional to the likelihood of each character state for a given ancestral node).

Using Blomberg’s *K* as our measure of phylogenetic signal, the LC50_4-d_ and LC50_8-d_ estimates both had values of *K* between zero and one, indicating some phylogenetic signal but less than expected under Brownian motion (i.e., partial phylogenetic dependence; Fig. 3). The signal observed for the LC50_4-d_ values was significant on the tree estimated from equal-length branches (*K* = 0.443, *P* = 0.034) but not on the tree generated from mitochondrial data (*K* = 0.552, *P* = 0.164). The signal observed for the LC50_8-d_ values was significant regardless of which tree was used (equal length *K* = 0.458, *P* = 0.042; mitochondrial data *K* = 0.743, *P* = 0.033). In general, bufonids were less sensitive than hylids and hylids were less sensitive than the ranids.

The phylogenetic analysis of the time lag showed five evolutionary transitions on the tree based on mtDNA (Fig. 3) and six transitions on the tree based on equal-length branches. In comparison, the randomized data showed an average of 10.39 and 10.35 transitions on each tree, respectively. This difference was highly significant for both trees, with a *P*-value of 0.003 (i.e., only in 3 of 1000 randomizations did we observed five or fewer evolutionary steps) on the mtDNA tree and a *P*-value of 0.007 on the equal-length-branches tree. Mortality time lags were common in ranids, occasional in hylids, and rare in bufonids.

## Discussion

On the basis of current EPA categories (Carey et al. 2008), endosulfan can be categorized as ‘very highly toxic’ to all eight species examined in this study (LC50_4-d_ and LC50_8-d_ < 100 ppb). Moreover, in half of the species, survival after a 4-day exposure was not indicative of survival after another 4 days in clean water. Most interestingly, when combined with the seven species from Jones et al. (2009), we found that the species-level variation in sensitivity to endosulfan contains a phylogenetic signal.

Endosulfan shows similar highly toxic effects to other organisms. Among the few tests that have been conducted on other amphibians, LC50_4-d_ estimates range from 1.8 to 4700 ppb across a variety of *Bufo* and *Rana* species (Gopal et al. 1981; Vardia et al. 1984; Harris et al. 1998b; Bernabò et al. 2008). Studies of fish and invertebrates have similar LC50 estimates: *Morone saxatilis* (striped bass) *Oncorhynchus mykiss* (rainbow trout), and *Lepomis macrochirus* (bluegill sunfish) range from 0.1–1.7 ppb; *Gammarus palustris* and *Hyalella azteca* (amphipods) range from 0.4 to 5.7 ppb (Leight and Van Dolah 1999; Wan et al. 2005); and *Procambarus clarkii* (red swamp crayfish) has an estimate of 120 ppb (Leight and Van Dolah 1999). The general pattern is that endosulfan is highly toxic (1000 ppb >LC50 > 100 ppb) to very highly toxic (LC50 < 100 ppb) for most aquatic organisms.

When estimating lethal concentrations, it is important to determine how the estimates compare to EEC. Estimates from wind-blown overspray indicate that aqueous endosulfan concentrations can range from 700 ppb at 10 m from the target site to 4 ppb at 200 m from the target site (Ernst et al. 1991). Actual endosulfan concentrations found in ponds near apple orchards in Ontario, Canada, were 0.53 ppb (Harris et al. 1998a) and reached 2.5 ppb in the Gwydir River basin in Australia (Muschal 2001). A recent model by the US EPA (2002) estimated the EEC of surface drinking water at 4.5–23.9 ppb for acute exposures and 0.5–1.5 ppb for chronic exposures. Collectively, these estimates suggest that tadpoles and many other aquatic organisms will often be exposed to endosuflan concentrations that cause substantial mortality.

How much mortality would we predict to occur at EEC among the 15 species of tadpoles considered in this study? Using the EPA chronic exposure and our LC50_8-d_ estimates, exposed populations would experience approximately 10% mortality in six of the tadpole species (*R. sphenocephala*, *R. sylvatica*, *P. regilla*, *H. versicolor*, *P. crucifer*, and *R. clamitans*) and 50% mortality in one tadpole species (*R. catesbeiana*; Jones et al. 2009). Using EPA acute exposure and our LC50_8-d_ estimates, exposed populations would experience approximately 10% mortality in three tadpole species (*P. triseriata*, *B. americanus*, and *B.* *boreas*), 50% mortality in six tadpole species (*R. pipiens*, *P. crucifer*, *P. feriarum*, *R. sylvatica*, *P. regilla*, and *H. versicolor*), and 90% mortality in six tadpole species (*R. cascadae*, *R. boylii*, *R. clamitans*, *R. aurora*, *R. sphenocephala*, and *R. catesbeiana*). In short, the estimated EEC values for endsulfan suggest that a large proportion of exposed amphibians would die from direct toxicity. Of course, the true effect of endosulfan exposure on amphibian populations in nature is difficult to estimate because of the additional stressors including competition, predation, and disease, and whether mortality from endosulfan would be additive or compensatory with other causes.

The lag effects of endosulfan observed in the current study appear to be a common phenomenon. Previous laboratory experiments by Berrill et al. (1998) found mortality increased dramatically after a single exposure to endosulfan over a 4-day period when *B. americanus*, *R. sylvatica*, and *R. clamitans* tadpoles were transferred to clean water for an additional 5–10 days. Although the concentrations (three between 40 and 365 ppb per species) and postexposure periods differed among the three species, mortality increased from 10% during the 4-day exposure period to 30% to 100% by the end of the postexposure period. In a mesocosm experiment, *R. pipiens* tadpoles exposed to endosulfan experienced 84% mortality, but this mortality took several days to develop, again suggesting a lag effect (Relyea 2009). Collectively, these studies suggest that the endosulfan continues to cause mortality well after direct exposure in many species of amphibians.

The lag effect in LC50 estimates presents a serious applied problem. While the mechanism underlying the lag effect is not known, the existence of the phenomenon means that traditional LC50 studies will underestimate endosulfan’s lethality to amphibians and potential other species. In *P. crucifer*, for example, the LC50_8-d_ value is approximately one-fourth the concentration of the LC50_4-d_ value. Plainly stated, the concentration that will kill 50% of the tadpoles is one-fourth the concentration we would estimate from a traditional LC50_4-d_ test. Given that regulatory agencies such as the US EPA set the ‘Level of Concern (LOC)’ for pesticides in nature at 5% or 10% of an animal’s LC50 estimate (for listed and nonlisted species, respectively), the existence of lag effects means that the LOC would be set four times too high for species such as *P. crucifer*. Incorporation of lag effects into standard toxicity test will provide more accurate estimates of environmental impacts for some pesticides.

Past studies of endosulfan effects on larval amphibians have noted substantial differences in sensitivities among species. The current study represents the first phylogenetic analysis of amphibian sensitivity to a pesticide and confirms previous suggestions of phylogenetic patterns for sensitivity to the insecticide endosulfan (Berrill et al. 1998; Jones et al. 2009; Relyea 2009). We found the phylogenetic signal for the LC50_4-d_ and LC50_8-d_ estimates exhibit similar patterns. In general, bufonids were the least sensitive group, followed by the hylids, which show large amounts of variation, and the ranids, which are generally highly sensitive. The ranid exceptions are *R. pipiens* and *R. sylvatica*, which were the least sensitive species of ranids and had LC50_8-d_ estimates that were similar to *P. crucifer* and *P. feriarum*. The mechanism driving these patterns is currently unknown, but it is likely linked to some aspect of conserved physiology within each clade, given that the insecticide has only been in use for the past 57 years.

Other pesticides show different patterns when tested across multiple species. For example, Relyea and Jones (2009) compared glyphosate sensitivity for nine species of anurans and four species of salamanders and found little variation between species (LC50_4-d_ for anurans = 0.8–2.0 ppm, LC50_4-d_ for salamanders = 2.7–3.2 ppm). Even without a formal phylogenetic analysis, it appears that glyphosate sensitivity has no phylogenetic signal. Interestingly, those nine anuran species were also assayed in the current study and show large amounts of variation in their sensitivity to endosulfan (LC50_4-d_ 1.3–112 parts per billion). The insecticide carbaryl also shows substantial interspecific variation within the ranids (Bridges and Semlitsch 2000). However, when ten populations of *R. sphenocephala* were assayed for population-level variation, the intraspecific variation was larger than the interspecific variation (Bridges and Semlitsch 2000). This high level of intraspecific variation creates inference problems because any single population may not reflect the species’ mean and any likely phylogenetic signal is a product of the specific populations sampled not necessarily a species-level difference.

We found little intraspecific variation among the populations of *R. sylvatica* exposed to endosulfan. The six populations all showed similar sensitivities, with only the least (LC50_2-d_ = 23.5 ppb) and most sensitive (LC50_2-d_ = 13.2 ppb) being significantly different. Endosulfan is commonly, though not heavily, used in Pennsylvania and Michigan (United States Geological Survey http://water.usgs.gov/nawqa/pnsp/usage/maps). These populations potentially have different exposure histories, yet still have similar sensitivities. More work though needs to be done on pesticides assaying population differences in general and specifically across historic pesticide use gradients.

The endosulfan LC50_4-d_ and LC50_8-d_ estimates exhibited phylogenetic signal, but less signal than expected under a Brownian motion model of character evolution (i.e., a value of *K* <1; Blomberg et al. 2003). The evolutionary implications of partial phylogenetic dependence or weak phylogenetic signal have not been well explored in the literature. Previous studies have shown that behavioral traits and group traits such as population density and geographic range size tend to show phylogenetic signal that is weaker than observed in highly heritable traits under strong selection such as body mass or fecundity (Blomberg et al. 2003; P. R. Stephens and J. L. Gittleman unpublished data). LC50 estimates are hard to classify as being either heritable or as products of either strong or weak selection because it depends on whether the specific population shares an exposure history with the pesticide. If the population has no exposure history, the LC50 estimate should be heavily influenced by ecological factors and phylogeny. However, if the population has been repeated exposed to high concentrations, the LC50 estimate can also be influenced by selection. Unfortunately for most populations, past and current exposure histories are unknown.

Exposure histories can become even more confounding when comparing among distantly related species because of propagation of variation with each species’ estimate, potentially not reflecting a true mean. However, many of these species co-occur and hence share a common exposure history. For example, *B. americanus* and *R. clamitans* were collected from the same pond as were *P. crucifer* and *H. versicolor*. Despite living in the same location and having potentially overlapping tadpole stages, these species have very different sensitivities to endosulfan.

The demonstration of phylogenetic patterns in sensitivity to endosulfan and time lags in mortality represents a critical step in our ability to predict the impact of the pesticide. For example, our results demonstrate that more closely related species share similar levels of sensitivity. Thus, at least for endosulfan, common closely related species may be used as surrogates for species of conservation concern. Furthermore, large-scale comparisons of other traits such as habitat use or disease tolerance can now be compared with pesticide sensitivity in a more rigorous manner using the same comparative methods that have been used to investigate patterns of evolutionary diversification in ecological traits (e.g., Winermiller 1991; Richman and Price 1992; Schluter 1994; Losos 1996; Losos et al. 1998; McPeek and Brown 2000; Richardson 2001; Stephens and Wiens 2004). These results argue that more research from a phylogenetic perspective on pesticide sensitivity is likely to generate key insights into the diversification of ‘resistance’ traits in amphibians and to have major implications for amphibian conservation.

## Data archiving statement

Data for this manuscript are available in Dryad: doi:10.5061/dryad.q2mf26f1.

## Appendix A1: The assembly of phylogenetic trees

The first tree was assembled by hand in Mesquite (Maddisson and Maddison 2010). Relationships among species of *Pseudacris* and *Hyla* were based on the studies of Wiens et al. (2006) and Moriarty and Cannatella (2004), relationships among species of *Rana* were based upon Wiens et al. (2009), and relationships among genera were based upon Hoegg et al. (2004). A speciational model of evolution was used to assign branch lengths to this tree, with all branches assigned a length of 1.

A second tree and branch lengths were also directly estimated for these species using a 2500 base pair region of mitochondrial sequence data that includes 12S, 16S, and adjacent transfer RNAs obtained from GenBank (http://ncbi.nlm.nih.gov/Genbank/index.html). Table A1 contains a list of accession numbers and sequence sources. Initial sequence alignments were generated using MUSCLE v3.8.31 (Edgar 2004), with some adjustments made by eye and regions of uncertain alignment excluded using Se-Al 2.0 (Rambaut 2008). Analyses of sequences were performed using RAxML v6.0.0 (Stamatakis et al. 2008), using maximum likelihood and a GTR model (general time reversible) model of sequence evolution. This is the only model of sequence evolution implemented in RAxML, and so no model fitting procedures were performed. Based on Hoegg (2004) and Wiens et al. (2009) two species of *Heleophryne*, *H. regis* and *H. purcelli*, were used as the outgroup to the other genera during analysis. The topology of the tree obtained using maximum likelihood was identical to that constructed based on previously published studies, save that the positions of *Pseudacris crucifer* and *P. regilla* were reversed (Fig A1).

**Table A1:** GenBank accession numbers of sequences used for phylogenetic analyses

Accession number	Scientific name	Reference
DQ283180	*Bufo boreas*	Frost et al. 2005^1^
AY680211	*Bufo americanus*	Pauly et al. 2004^2^
AY843738	*Pseudacris triseriata*	Faivovich et al. 2005^3^
EF472219	*Pseudacris feriarum*	Lemmon et al. 2007^4^
AY843737	*Pseudacris regilla*	Faivovich et al. 2005^3^
AY843735	*Pseudacris crucifer*	Faivovich et al. 2005^3^
AY843682	*Hyla versicolor*	Faivovich et al. 2005^3^
DQ283123	*Rana pipiens*	Frost et al. 2005^1^
AY779251	*Rana sphenocephala*	Hillis and Wilcox 2005^5^
DQ283387	*Rana sylvatica*	Frost et al. 2005^1^
DQ283185	*Rana clamitans*	Frost et al. 2005^1^
DQ283257	*Rana catesbeiana*	Frost et al. 2005^1^
DQ283189	*Rana aurora*	Frost et al. 2005^1^
AY779197	*Rana cascadae*	Hillis and Wilcox 2005^5^
AY779192	*Rana boylii*	Hillis and Wilcox 2005^5^
DQ283115.1	*Heleophryne regis*	Frost et al. 2005^1^
AY843593.1	*Heleophryne purcelli*	Faivovich et al. 2005^3^

1 Frost, D. R., T. Grant, J. Faivovich, R. Bain, A. Haas, C. F. B. Haddad, R. O. de Sa, A. Channing, M. Wilkinson, S. C. Donnellan, C. Raxworthy, J. A. Campbell, B. L. Blotto, P. Moler, R. C. Drewes, R. A. Nussbaum, J. D. Lynch, D. M. Green, and W. C. Wheeler 2006. The Amphibian Tree of Life. *Bulletin of the American Museum of Natural History***297:** 1–291.

2 Pauly, G. B., D. M. Hillis and D. C. Cannatella 2004. The history of a nearctic colonization: molecular phylogenetics and biogeography of the Nearctic toads (*Bufo*). *Evolution***58:** 2517–2535.

3 Faivovich, J., C. F. B. Haddad, P. C. A. Garcia, D. R. Frost, J. A. Campbell, and W. C. Wheeler 2005. Systematic Review of the frog family Hylidae, with special reference to the Hylinae: Phylogenetic analysis and taxonomic revision. *Bulletin of the American Museum of Natural History***294:** 1–240.

4 Lemmon, E. M., A. R. Lemmon, J. T. Collins, J. A. Lee-Yaw, and D. C. Cannatella 2007. Phylogeny-based delimitation of species boundaries and contact zones in the trilling chorus frogs (*Pseudacris*). *Molecular Phylogenetics and Evolution***44:** 1068–1082.

5 Hillis, D. M. and T. P. Wilcox. 2005. Phylogeny of the New World true frogs (Rana). *Molecular Phylogenetics and Evolution***34:** 299–314.

## Appendix A2: Single Species data

*Bufo americanus* survival in the water and vehicle controls was 100% after both 4 and 8 days (Fig. 1). After 4 days, the 20, 38, 162 ppb concentrations experienced significantly more death than the control. After 8 days, the same endosulfan concentrations were significantly different from the control. Based on these results, the LC50 estimates were 55.5 ppb after 4-days, and 53.0 ppb after an additional 4-days in clean water (Table 1). Endosulfan had no significant lag effect on mortality of *B. americanus*.

In *P. triseriata*, survival in the water and vehicle controls was 100% after both 4 and 8 days (Fig. 1). After 4 days, only the 109 ppb concentration had significantly more death than the control. After 8 days, the 109 ppb endosulfan concentration was still the only concentration significantly different from the control. The LC50 estimates were 56.4 ppb after 4 days and 53.6 ppb after an additional 4 days in clean water (Table 1). Based on these data, endosulfan had no significant lag effect on mortality of *P. triseriata*.

For *P. feriarum*, survival in the water and vehicle controls was initially very good but declined over time (100% and 92% after 4 days; 82% and 82% after 8 days, respectively; Fig. 1). After 4 days, the 14, 18, and 109 ppb concentrations exhibited significantly greater mortality than the control. After 8 days, only the 109 ppb concentration was different from the control because of the control mortality adjustment. Based on these results, the LC50 estimates were 25.5 ppb after 4 days and 25.3 ppb after an additional 4 days in clean water (Table 1). Endosulfan had no significant lag effect on mortality of *P. feriarum*.

For *R. pipiens* survival was excellent in the water and vehicle controls (100% and 100% after 4 days; 100% and 98% after 8 days, respectively; Fig. 1). After 4 days, the 20, 38, and 162 ppb concentrations caused significantly more death than the control. After 8 days, the 3, 20, 38, and 162 concentrations were significantly different from the control. Based on these data, the LC50 estimates were 51.0 ppb after 4 days of endosulfan exposure and 28.0 ppb after an additional 4 days in clean water (Table 1). *R. pipiens* tadpoles experienced a significant lag effect to endosulfan exposure.

*Rana sphenocephala*, survival in the water and vehicle controls was excellent (100% after both 4 and 8 days; Fig. 1). After 4 days, the 2, 3, 20, 38, and 162 ppb concentrations caused significantly more death than the control. After 8 days, the same concentrations again were significant different from the control. The LC50 estimate was 2.8 ppb after 4 days, but 1.9 ppb after an additional 4 days in clean water, which is significantly lower based on the 84% confidence intervals (Table 1). *R. sphenocephala* mortality exhibited a small lag effect following exposure to endosulfan.

*Rana sylvatica* survival in the water and vehicle controls was high over the first 4 days (93% and 95% respectively) but declined over the following 4 days (85% and 83%; Fig. 1). After the first 4 days, concentrations of 3, 38, and 162 (but not 20) ppb caused significantly more death than the control. After 8 days, only the 38 and 162 ppb concentrations were significantly different from the control. The LC50 estimates were 31.4 ppb after 4 days and 25.1 ppb after 8 days (Table 1). Based on these data, *R. sylvatica* experience no significant lag effect.

*Rana aurora* also experienced high survival in the water and vehicle controls (97% and 100% after 4 days; 93% and 97% after 8 days, respectively; Fig. 1). After 4 days, the 20, 38, and 162 ppb concentrations had significantly more death than the control. After 8 days, the 3, 20, 38, and 162 ppb concentrations had significantly more death than the control. The LC50 estimate was 3.7 ppb after 4 days and 2.4 ppb after an additional 4 days in clean water (Table 1). Based on these data, *R. aurora* mortality exhibited a small lag effect following exposure to endosulfan.

For *R. boylii*, survival in the water and vehicle controls was 100% after both 4 and 8 days (Fig. 1). After 4 days, tadpoles exposed to 3, 16, 38, and 145 ppb of endosulfan had significantly greater death than the control. After 8 days, 2, 3, 16, 38, and 145 ppb concentrations were significantly from the control. Using these data, the LC50 estimates were 13.0 ppb after 4 days and 5.4 ppb after four more days in clean water (Table 1). Based on these data, *R. boylii* experienced a lag effect of endosulfan exposure.

**Table A2:** The species of tadpoles used in the endosulfan experiments including common and family name, number of egg masses, initial mass(mean ± 1 SE), and developmental stage. Underlined species are estimates from a previous study (Jones et al. 2009)

Scientific name	Common name	Family	Egg masses	Mass (mg)	Gosner stage
*Bufo boreas*	Western toad	Bufonidae	10	63 ± 3	26
*Bufo americanus*	American toad	Bufonidae	12	38 ± 2	27
*Pseudacris triseriata*	Western chorus frog	Hylidae	10	53 ± 3	29
*Pseudacris feriarum*	Southeastern chorus frog	Hylidae	20	52 ± 3	29
*Pseudacris regilla*	Pacific tree frog	Hylidae	10	83 ± 3	26
*Pseudacris crucifer*	Spring peeper	Hylidae	28	27 ± 3	26
*Hyla versicolor*	Gray tree frog	Hylidae	30	59 ± 3	26
*Rana pipiens*	Northern leopard frog	Ranidae	10	44 ± 2	25
*Rana sphenocephala*	Southern leopard frog	Ranidae	8	41 ± 2	25
*Rana sylvatica*	Wood frog	Ranidae	10	48 ± 2	26
*Rana clamitans*	Green frog	Ranidae	15	44 ± 3	25
*Rana catesbeiana*	American bullfrog	Ranidae	15	40 ± 6	25
*Rana aurora*	Red-legged frog	Ranidae	13	56 ± 3	25
*Rana cascadae*	Cascades frog	Ranidae	3	63 ± 4	27
*Rana boylii*	Foothill yellow-legged frog	Ranidae	8	45 ± 2	25

**Table A3:** The populations of *R. sylvatica* used in the population endosulfan experiment including number of egg masses, initial mass (mean ± 1 SE), and developmental stage

Population	Egg masses	Mass (mg)	Gosner stage
1	10	158 ± 10	28
2	10	221 ± 22	30
3	10	144 ± 8	28
4	10	172 ± 17	28
5	9	243 ± 17	29
6	7	157 ± 8	28

## References

[b1] Alford RA, Richards SJ (1999). Global amphibian declines: a problem in applied ecology. Annual Review of Ecology and Systematics.

[b2] Ashauer R, Hinermeister A, Potthoff E, Escher BI (2011). Acute toxicity of organic chemicals to *Gammarus pulex* correlates with sensitivity of *Daphnia magna* across most modes of action. Aquatic Toxicology.

[b3] Bernabò I, Brunelli E, Berg C, Bonacci A, Tripepi S (2008). Endosulfan acute toxicity in *Bufo bufo* gills: ultrastructural changes and nitric oxide synthase localization. Aquatic Toxicology.

[b4] Berrill M, Coulson D, McGillivray L, Pauli B (1998). Toxicity of endosulfan to aquatic stages of anuran amphibians. Environmental Toxicology and Chemistry.

[b5] Blaustein AR, Kiesecker adJM (2002). Complexity in conservation: lessons from the global decline of amphibian populations. Ecology Letters.

[b6] Blomberg SP, Garland T (2002). Tempo and mode in evolution: phylogenetic inertia, adaptation and comparative methods. Journal of Evolutionary Biology.

[b7] Blomberg SP, Garland T, Ives AR (2003). Testing for phylogenetic signal in comparative data: behavioral traits are more labile. Evolution.

[b8] Boone MD, Semlitsch RD, Little EE, Doyle MC (2007). Multiple stressors in amphibian communities: effects of chemical contamination, bullfrogs, and fish. Ecological Applications.

[b9] Bridges CM, Semlitsch RD (2000). Variation in pesticide tolerance of tadpoles among and within species of Ranidae and patterns of amphibian decline. Conservation Biology.

[b10] Carey S, Crk T, Flaherty C, Hurley P, Hetrick J, Moore K, Termes SC (2008). Risks of Glyphosate Use to Federally Threatened California Red-Legged Frog (Rana aurora draytonii).

[b12] Ernst WR, Jonah P, Doe K, Julien G, Hennigar P (1991). Toxicity to aquatic organisms of off-target deposition of endosulfan applied by aircraft. Environmental Toxicology and Chemistry.

[b13] Felsenstein J (1973). Maximum likelihood and minimum-step methods for estimating evolutionary tress from data on discrete characters. Systematic Zoology.

[b14] Finney DJ (1971). Probit Analysis.

[b16] Gilliom RJ, Hamilton PA (2006). Pesticides in the Nation’s Streams and Ground Water, 1992–2001 – A Summary. Fact Sheet.

[b17] Gopal K, Khanna RN, Anand M, Gupta GSD (1981). The acute toxicity of endosulfan to fresh-water organisms. Toxicology Letters.

[b18] Gosner KL (1960). A simplified table for staging anuran embryos and larvae with notes on identification. Herpetologica.

[b20] Harris ML, Bishop CA, Struger J, Van Den Heuvel MR, Van Der Kraak GJ, Dixon DG, Ripley B (1998a). The functional integrity of northern leopard frog (*Rana pipiens*) and green frog (*Rana clamitans*) populations in orchard wetlands. I. Genetics, physiology, and biochemistry of breeding adults and young-of-the-year. Environmental Toxicology and Chemistry.

[b21] Harris ML, Bishop CA, Struger J, Ripley B, Bogart JP (1998b). The functional integrity of northern leopard frog (*Rana pipiens*) and green frog (*Rana clamitans*) populations in orchard wetlands. II. Effects of pesticides and eutrophic conditions on early life stage development. Environmental Toxicology and Chemistry.

[b22] Harris ML, Chora L, Bishop CA, Bogart JP (2000). Species- and age-related differences in susceptibility to pesticide exposure for two amphibians, *Rana pipiens,* and *Bufo americanus*. Bulletin of Environmental Contamination and Toxicology.

[b23] Hayes TB, Collins A, Lee M, Mendoza M, Noriega M, Stuart AA, Vonk A (2002). Hermaphroditic, demasculinized frogs after exposure to the herbicide atrazine at low ecologically relevant doses. Proceedings of the National Academy of Sciences of the United States of America.

[b24] Hayes TB, Case P, Chui S, Chung D, Haefele C, Haston K, Lee M (2006). Pesticide mixtures, endocrine disruption, and declines: are we underestimating the impact?. Environmental Health Perspectives.

[b26] Hopkins WA, Mendonca MT, Rowe CL, Congdon JD (1998). Elevated trace element concentrations in southern toads, *Bufo terrestris*, exposed to coal combustion waste. Archives of Environmental Contamination and Toxicology.

[b27] Jones R, Leahy J, Mahoney M, Murray L, Odenkirchen E, Petrie R, Stangel C (2004). Overview of the Ecological Risk Assessment Process in the Office of Pesticide Programs, U.S. Environmental Protection Agency.

[b28] Jones DK, Hammond JI, Relyea RA (2009). Very highly toxic effects of endosulfan across nine species of tadpoles: lag effects and family-level sensitivity. Environmental Toxicology and Chemistry.

[b29] Jones DK, Hammond JI, Relyea RA (2010). Roundup® and amphibians: the importance of concentration, application time, and stratification. Environmental Toxicology and Chemistry.

[b30] Kembel S, Cowan P, Helmus M, Cornwell W, Morlon H, Ackerly D, Blomberg S (2010). Picante: R tools for integrating phylogenies and ecology. Bioinformatics.

[b31] Leight AK, Van Dolah RF (1999). Acute toxicity of the insecticides endosulfan, chlorpyrifos, and malathion to the epibenthic estuarine amphipod *Gammarus palustris* (Bousfield). Environmental Toxicology and Chemistry.

[b32] LeNoir JS, McConnell LL, Fellers GM, Cahill TM, Seiber JN (1999). Summertime transport of current-use pesticides from California’s central valley to the Sierra Nevada mountain range, USA. Environmental Toxicology and Chemistry.

[b33] Losos JB (1996). Phylogenetic perspectives on community ecology. Ecology.

[b34] Losos JB, Jackman TR, Larson A, de Queiroz K, Rodriguez-Schettino L (1998). Contingency and determinism in replicated adaptive radiations of island lizards. Science.

[b36] Maddison WP, Slatkin M (1991). Null models for the number of evolutionary steps in a character on a phylogenetic tree. Evolution.

[b37] McConnell LL, LeNoir JS, Datta S, Seiber JN (1998). Wet deposition of current-use pesticides in the Sierra Nevada mountain range, California, USA. Environmental Toxicology and Chemistry.

[b38] McPeek MA, Brown JM (2000). Building a regional species pool: diversification of the Enallagma damselflies in eastern North America. Ecology.

[b40] Muschal M (2001). Central & North West Region's Water Quality Program. CNR2000.067. CNWRWQP Pesticide Report.

[b41] Newman RA, Squire T (2001). Microsatellite variation and fine-scale population structure in wood frog (*Rana sylvatica*. Molecular Ecology.

[b42] Pagel M (1999). Inferring the historical patterns of biological evolution. Nature.

[b45] Payton ME, Greenstone MH, Schenker N (2003). Overlapping confidence intervals or standard error intervals: what do they mean in terms of statistical significance?. Journal of Insect Science.

[b47] Relyea RA (2002). Local population differences in phenotypic plasticity: predator-induced changes in wood frog tadpoles. Ecological Monographs.

[b48] Relyea RA (2009). A cocktail of contaminants: how mixtures of pesticides at low concentrations affect aquatic communities. Oecologia.

[b49] Relyea RA, Diecks N (2008). An unforeseen chain of events: lethal effects of pesticides at sublethal concentrations. Ecological Applications.

[b50] Relyea RA, Hoverman JT (2008). Interactive effects of predators and a pesticide on aquatic communities. Oikos.

[b51] Relyea RA, Jones DK (2009). The toxicity of roundup original max® to 13 species of larval amphibians. Environmental Toxicology and Chemistry.

[b52] Richardson JML (2001). The relative roles of adaptation and phylogeny in determination of larval traits in diversifying anuran lineages. American Naturalist.

[b53] Richman AD, Price T (1992). Evolution of ecological differences in the old-world leaf warblers. Nature.

[b54] Rohr JR, Crumrine PW (2005). Effects of an herbicide and an insecticide on pond community structure and processes. Ecological Applications.

[b55] Rohr JR, Schotthoefer AM, Raffel TR, Carrick HJ, Halstead N, Hoverman JT, Johnson CM (2008). Agrochemicals increase trematode infections in a declining amphibian species. Nature.

[b56] Rossi LA (2002). Reregistration Eligibility Decision for Endosulfan.

[b57] Schluter D (1994). Experimental-evidence that competition promotes divergence in adaptive radiation. Science.

[b58] Schluter D, Price T, Mooers AØ, Ludwig D (1997). Likelihood of ancestor states in adaptive radiation. Evolution.

[b59] Sparling DW, Fellers GM (2007). Comparative toxicity of chlorpyrifos, diazinon, malathion, and their oxon derivatives to larval *Rana boylii*. Environmental Pollution.

[b60] Sparling DW, Fellers GM, McConnell LL (2001). Pesticides and amphibian population declines in California, USA. Environmental Toxicology Chemistry.

[b62] Stephens PR, Wiens J (2004). Convergence, divergence, and homogenization in the ecological structure of emydid turtle communities: the effects of phylogeny and dispersal. American Naturalist.

[b63] Strumbaurer C, Opadiya GB, Niederstätter H, Riedmann A, Dallinger R (1999). Mitochondrial DNA reveals cryptic oligochaete species differing in cadmium resistance. Molecular Biology and Evolution.

[b64] Taylor SK, Williams ES, Mills KW (1999). Effects of malathion on disease susceptibility in Woodhouse’s toad. Journal of Wildlife Disease.

[b65] Vardia HK, Rao PS, Durve VS (1984). Sensitivity of toad larvae to 2,4-D and endosulfan pesticides. Archiv fuer Hydrobiologie.

[b66] Wan MT, Kuo J, Buday C, Schroeder G, Van Aggelen G, Pasternak J (2005). Toxicity of α, β-, (α + β)-endosulfan and their formulated and degradation products to *Daphnia magna**Hyalella azteca**Oncorhynchus mykiss**Oncorhynchus kisutch*, and biological implications in streams. Environmental Toxicology and Chemistry.

[b69] Winermiller KO (1991). Ecomorphological diversification in lowland fresh-water fish assemblages from 5 biotic regions. Ecological Monographs.

